# Ageing causes an aortic contractile dysfunction phenotype by targeting the expression of members of the extracellular signal‐regulated kinase pathway

**DOI:** 10.1111/jcmm.17118

**Published:** 2022-02-18

**Authors:** Christopher J. Nicholson, Yi Xing, Sophie Lee, Stephanie Liang, Shivani Mohan, Caitlin O’Rourke, Joshua Kang, Kathleen G. Morgan

**Affiliations:** ^1^ Department of Health Sciences Boston University Boston MA USA; ^2^ Department of Medicine Cardiology Division Cardiovascular Research Center Massachusetts General Hospital Harvard Medical School Boston MA USA

**Keywords:** ageing, focal adhesion, microRNA, thoracic aortic aneurysm, vascular smooth muscle cell

## Abstract

The extracellular signal‐regulated kinase (ERK) pathway is a well‐known regulator of vascular smooth muscle cell proliferation, but it also serves as a regulator of caldesmon, which negatively regulates vascular contractility. This study examined whether aortic contractile function requires ERK activation and if this activation is regulated by ageing. Biomechanical experiments revealed that contractile responses to the alpha1‐adrenergic agonist phenylephrine are attenuated specifically in aged mice, which is associated with downregulation of ERK phosphorylation. ERK inhibition attenuates phenylephrine‐induced contractility, indicating that the contractile tone is at least partially ERK‐dependent. To explore the mechanisms of this age‐related downregulation of ERK phosphorylation, we transfected microRNAs, miR‐34a and miR‐137 we have previously shown to increase with ageing and demonstrated that in A7r5 cells, both miRs downregulate the expression of Src and paxillin, known regulators of ERK signalling, as well as ERK phosphorylation. Further studies in aortic tissues transfected with miRs show that miR‐34a but not miR‐137 has a negative effect on mRNA levels of Src and paxillin. Furthermore, ERK phosphorylation is decreased in aortic tissue treated with the Src inhibitor PP2. Increases in miR‐34a and miR‐137 with ageing downregulate the expression of Src and paxillin, leading to impaired ERK signalling and aortic contractile dysfunction.

## 1 INTRODUCTION

It is well established that vascular tone is increased by Ca^2+^/Calmodulin‐dependent myosin light chain kinase (MLCK) activation, which promotes myosin phosphorylation and actomyosin cross‐bridge cycling.^1^ However, the ERK‐dependent removal of actin inhibition by caldesmon (CaD) can also increase cross‐bridge cycling velocity and smooth muscle contractility.^2^, ^3^, ^4^ In smooth muscle cells, the PKC‐dependent activation of Raf and MEK activates ERK1/2 through tyrosine/threonine phosphorylation.^5^ In contractile smooth muscle ERK activation results in the downstream phosphorylation and inactivation of the inhibitory actin‐binding protein CaD, which normally functions to block the access of myosin to actin and hence impair cross‐bridge cycling, much like troponin does in striated muscle.^6^, ^7^, ^8^ Phosphorylation of CaD by ERK results in removal of this inhibitory effect and causes an increase in smooth muscle contractility. Previous studies have also demonstrated that ERK inhibitors attenuate contractility of the rat aorta^9^, ^10^, ^11^, ^12^, ^13^ but studies of the role of ageing on ERK activation and aortic function are lacking. Here, we focused on the widely used mouse model, the C75BL/6J mouse.

We recently demonstrated that miRs‐34a and −137 are upregulated with ageing in the mouse aorta.^14^ These miRs were predicted to target proteins involved in both ERK activation and ERK scaffolding, which play important roles in coordinating ERK signalling. We postulated, therefore, that these miRs may contribute to aortic smooth muscle contractile dysfunction with age and may represent novel targets to prevent thoracic aortic malfunction in the elderly. Thus, the purpose of the present study was to test the hypothesis that upregulation of miRs‐34a and −137 with ageing in the mouse model might cause a pathophysiologic change of the smooth muscle of aorta via an ERK‐dependent mechanism.

## 2 MATERIALS AND METHODS

All animal procedures were performed in accordance with the NIH Guide for the Care and Use of Laboratory Animals and according to protocols approved by the Institutional Animal Care and Use Committee of Boston University (Permit Number: A3316‐01) and used in compliance with federal, state and local laws.

### 2.1 Animals

Young adult C57Bl6 J mice (3months old) were purchased from Jackson Laboratories. Pre‐aged 24–28 months old mice were obtained from the NIA aged rodent colonies. Only tissues removed from animals euthanized in the Boston University Animal Science Center were used in this study. The animals were maintained according to the guidelines set out by the NIH Guide for the Care and Use of Laboratory Animals and were obtained and used in compliance with federal, state and local laws. Mouse aortas were quickly extracted immediately after death by euthanasia by isoflurane (Trade name: Forane) inhalation.

### 2.2 Preparation of aorta samples

After euthanization with an overdose of inhaled isofluorane in accord with institutional approval, #PROTO201900004, and in accord with the NIH guidelines, descending thoracic aortas were quickly excised from young (3–4 months) and aged (24–29 months) mice and kept in ice‐cold tissue‐collecting buffer (TCB; modified Krebs solution, in mM: 154 NaCl, 5.4 KCl, 1.2 MgSO_4_, 10 MOPS, 5.5 glucose and 1.6 CaCl_2_; pH = 7.4). Adipose tissue around the aortas was cleaned and each thoracic aorta was evenly cut into two rings in preparation for stress measurements. After stress measurements, for biochemical analyses, rings were quick‐frozen in freezing solution (10 mM dithiothreitol and 10% trichloroacetic acid dissolved in dry ice‐acetone mixture for western blot analysis), as described previously.^15^

### 2.3 Cell culture

A7r5 rat aortic smooth muscle cells (ATCC) were cultured in DMEM high glucose with 10% foetal bovine serum (FBS), 100 units/ml penicillin and 100 μg/ml streptomycin. Cells were grown to confluency before experiments and serum‐starved to drive them into a differentiated state similar to contractile smooth muscle cells 24 h before experimentation.^16^

### 2.4 Biomechanics

Aortic segments were mounted using two triangular wires (0.254‐mm diameter). Wall force was recorded by a force transducer attached to the upper triangle. The lower triangle was attached to a micrometer, which allowed adjustment of stretch during the normalization procedure. After mounting, aortic segments were incubated in organ baths containing warmed (37°C) oxygenated (95% O_2_–5% CO_2_) physiological salt solution (PSS; in mM: 120 NaCl, 5.9 KCl, 1.2 NaH_2_PO_4_, 25 NaHCO_3_, 11.5 dextrose, 2.5 CaCl_2_ and 1.2 MgCl_2_; pH = 7.4). Stretch was monitored by a 300C Dual‐Mode Lever Arm System from Aurora Scientific and force was recorded in Chart software (AD Instruments, Sydney). Before experimentation, each aortic segment was stretched to a previously determined optimal diameter for force production (1.8 × slack diameter) and left to equilibrate for 45 min. Vessel viability was confirmed by addition of PSS, in which 51 mM NaCl was replaced with KCl, for 15 min, followed by a return to PSS for 30 min. Segments that did not respond to KCl were discarded. Segments were then exposed to increasing doses (2 min duration each, of 10^−8^, 10^−7.5^, 10^−7^, 10^−6.5^, 10^−6^, 10^−5.5^, 10^−5 ^M) of the alpha1‐adrenoreceptor agonist phenylephrine (PE). Measurements of force generated were collected at baseline and at steady state after each dose. In a subset of experiments, segments were pre‐treated with the selective ERK inhibitor FR 180204 (10 µM, Tocris Biosciences) or vehicle control (H_2_O) for 30 min prior to experimentation.

Ex vivo aortic stress in this study was measured as the amplitude of force normalized to cross‐sectional area (CSA) using the equation: Stress = ∆F/(2DL), where ∆F is the amplitude of the force, D is the wall thickness, and L is the length of aortic strips.

### 2.5 Transfection of Aortic tissues

Each freshly dissected aorta was cut into 3 pieces (3 mm in length). Aortic tissues were transfected with 0.6% Lipofectamine RNAiMAX reagent mixed with Negative Control #1 (Control), hsa‐miR‐34a‐5p (miR‐34a) or hsa‐miR‐137‐3p (miR‐137) mirVana^™^ miRNA mimics for 48 h for RNA analysis. Control, miR‐34a and −137 miR mimics were diluted in Opti‐Mem medium at a final concentration of 100 nM. After 48 h‐incubation, biomechanical experiments were performed to test viability and contractility of aortic segments in response to 51 mM KCl solution and 10^−5 ^M PE. Aortic segments that responded to KCl solution were frozen at −80°C for further RNA analyses.

### 2.6 Transfection of cells

A7r5 cells were cultured in 6‐well plates and grown to confluency at a density of 2 × 10^5^ cells/well. Cells were transfected using 0.6% Lipofectamine RNAiMAX reagent mixed with control miR, miR‐34 or −137 mimics for either 24 h (for RNA analyses) or 72 h (for protein analyses). Control, miR‐34a and −137 miR mimics were diluted in Opti‐Mem medium at a final concentration of 10 nM. For the cell stimulation experiments, A7r5 cells were treated with the vasoconstrictor 12‐deoxyphorbol 13‐isobutylate 20‐acetate (DPBA) for 10 min. Since DPBA was diluted in dimethylsulphoxide (DMSO), equimolar DMSO (0.03%) was added to unstimulated cells as a vehicle control.

### 2.7 RNA quantification

RNA was isolated with a TRIzol‐based protocol at 4°C. Briefly, cells were scraped from the cell culture plates directly in TRIzol and then mixed several times. Aortic tissues were homogenized in TRIzol using the Precellys^®^ 24 tissue homogenizer (Bertin Technologies) and RNA was isolated with RNeasy Plus Micro Kit (QIAGEN). RNA concentration and quality were quantified by NanoDrop (Agilent Technologies). A 260/280 ratio of >1.75 was required for further study. RNA was reverse transcribed by the TaqMan Reverse Transcription kits. For mRNA analysis, TaqMan mouse gene expression assays were used for Pxn (Mm00448533_m1), Src (Mm00436785_m1) and Ptk2 (FAK, Mm00433209_m1). Gene expression was normalized to Gapdh (Mm99999915_g1). Quantitative reverse transcription polymerase chain reaction (qRT‐PCR) was performed on the StepOnePlus Real‐Time PCR system (Applied Biosystems). All fold changes were calculated by the ∆∆Ct method as described previously^17^ and compared with either young mice (in ageing comparisons) or scrambled control (in miR mimic comparisons).

### 2.8 Western blotting

A7r5 cells were scraped in lysis buffer (62.5 mM Tris, 2% SDS and 10% sucrose supplemented with protease inhibitor cocktail) and vortexed several times. Aortic tissues were homogenized in homogenization buffer (20 mM MOPS, 4% SDS, 10% glycerol, 10 mM DTT) supplemented with phosphatase and protease inhibitor using the tissue homeginizer. Cell lysates and tissue homogenates were cleared by centrifugation for 10 min at 13,000 rpm, 4°C. Fifteen µg of protein was resolved by SDS‐PAGE, transferred to a nitrocellulose membrane for western blotting with the following antibodies: ERK, paxillin, Src, FAK, p‐ERK1/2, or GAPDH and viewed following incubation with IRDye 680RD goat anti‐rabbit or IRDye 800CW goat anti‐mouse IgGs. Protein bands were visualized on an Odyssey infrared imaging system (LI‐COR) and densitometric analysis was performed with the Odyssey 2.1 software (LI‐COR). Intensity was adjusted for display purposes, but all quantitative analysis was performed on the raw data. For quantitative analysis of protein expression, bands of interest were normalized to GAPDH or total protein.

### 2.9 Reagents and antibodies

General laboratory reagents were obtained at analytic grade from Thermo Fisher Scientific, VWR International, Sigma‐Aldrich and Bio‐Rad. The alpha1‐adrenoreceptor agonist phenylephrine (PE) was purchased from Sigma‐Aldrich. Cell culture reagents were acquired from Invitrogen (Thermo Fisher Scientific) or ATCC. For the stimulation of A7r5 cells, DPBA (LC Laboratories) was used at a final concentration of 3 µM. The following primary antibodies were used: ERK (mouse, 1:500) and phospho‐ERK (rabbit, 1:1000) from Cell Signaling Technology; FAK (rabbit, 1:400) and Src (rabbit, 1:500) from Santa Cruz Biotechnology; Src (mouse, 1:500) from Abgent; paxillin (mouse, 1:500) from BD Biosciences; GAPDH (rabbit, 1:20,000) from Sigma‐Aldrich. Secondary antibodies used were IRDye^®^ 680 RD Goat anti‐Rabbit (1:1000) and IRDye^®^ 800CW Goat anti‐Mouse (1:1000) from LI‐COR Biosciences. RNA‐based experimental reagents, including TRIzol, Taqman and miR mimic kits were acquired from Life Technologies (Thermo Fisher Scientific).

### 2.10 Statistics

All values are presented as mean ± SEM. Analysis was carried out using GraphPad Prism (7.0) software. For biochemical analysis (of mRNA and protein expression) and stress comparisons, groups were compared using a Student's *t*‐test (two‐tailed) or two‐way ANOVA for parametric data. For grouped analyses, data were analysed with Tukey's multiple comparisons test. Significance was assumed at *p* < 0.05.

## 3 RESULTS

### 3.1 Contractility is downregulated in thoracic aortas of aged mice

**FIGURE 1:**
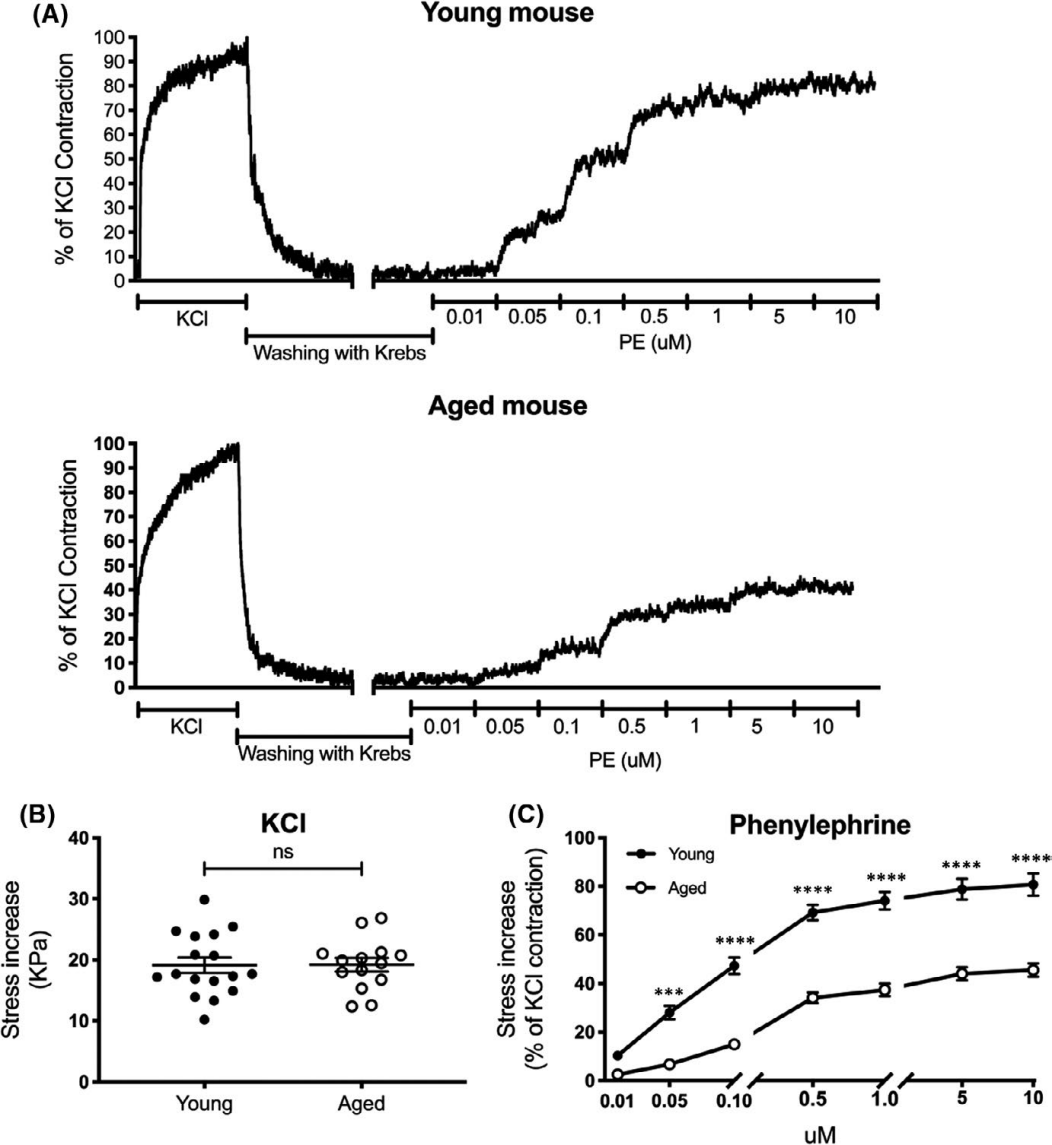
PE‐induced contraction is reduced in the thoracic aortas from aged mice. A, Representative raw force recordings from young (3‐month‐old) (top) and aged (28‐month‐old) (bottom) mouse aortas. B, Potassium chloride (KCl)–induced contractility was similar in isolated aortas from young (*n* = 17) and aged (*n* = 14) mice (two‐tailed Student's *t* test). C, PE‐induced aortic contractility (expressed as a percentage of KCl‐induced contractions) is attenuated with ageing (*n* = 21 young, 10 aged, two‐way ANOVA). ****p < 0.0001, ***p < 0.001

To determine the effects of ageing on thoracic aortic contractility, we exposed isolated aortic strips to either a depolarizing stimulus, 51 mM potassium chloride physiological saline solution (KCl) or incremental doses of the alpha1‐adrenergic receptor‐selective agonist phenylephrine (PE). Representative raw force tracings from young (Figure 1A, top) and aged (Figure 1A, bottom) aortic segments are displayed in Figure 1A. Segments were exposed to KCl, returned to PSS and then subjected to incremental doses of PE. Interestingly, there was no significant difference (*p *= 0.9646) in contractile responses to KCl between aortic strips from young and aged mice (Figure 1B). However, PE‐induced contractility was significantly impaired (*p *= 0.0005 for 0.05 μM; *p *< 0.0001 for 0.1 μM and higher concentrations) in proximal aortic strips from aged mice (Figure 1C).

### 3.2 Downregulation of PE‐induced ERK activation contributes to ageing‐related smooth muscle contractile dysfunction

While KCl‐induced contraction is calcium‐dependent, we and others have demonstrated that PE triggers an additional ERK signalling pathway that causes Ca^2+^ sensitization.^4^, ^18^ However, the role of ERK signalling has not yet been studied in the context of aortic contractility and ageing. Since KCl‐induced contractions did not differ between age groups, we postulated that aberrant ERK signalling may be responsible for the age‐related smooth muscle contractile dysfunction observed in Figure 1C.

**FIGURE 2:**
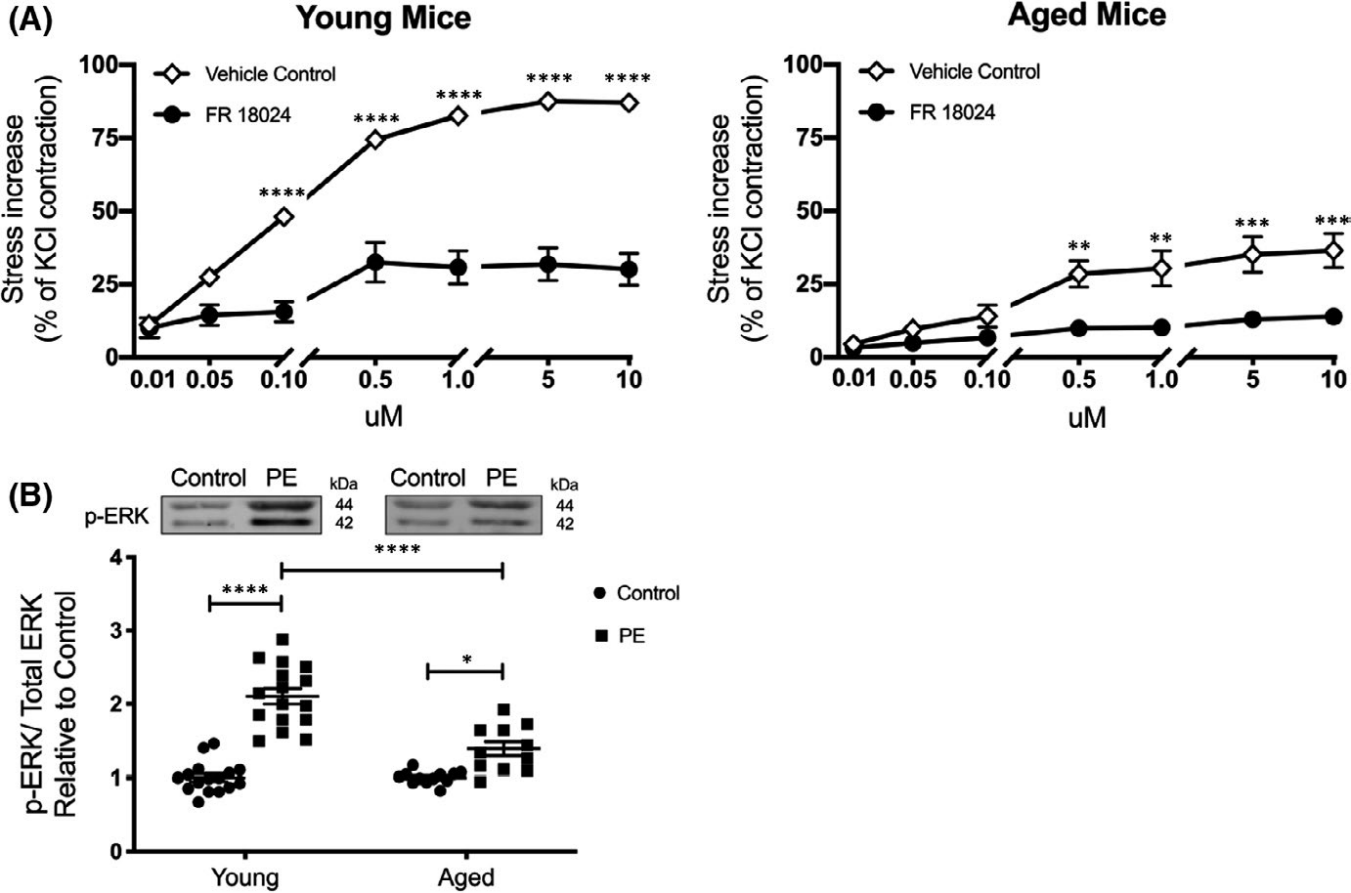
Ageing reduces phenylephrine‐induced ERK activation, which may contribute to smooth muscle contractile dysfunction. A, The use of an ERK inhibitor (FR 18,024, 10 µM) demonstrates that ERK activity is required for PE‐induced smooth muscle contraction in aortas from young (*n* = 5, left panel) and aged (*n* = 6 young 5 aged, right panel) mice. B, PE (10 µM for 10 min)‐induced ERK phosphorylation was higher in young (*n* = 16) compared to aged (*n* = 12) aortas. PE numbers normalized to controls. ****p < 0.0001, ***p < 0.001, **p < 0.01, *p < 0.05 (two‐way ANOVA)

We first sought to determine whether the ERK signalling pathway is required for contraction in the mouse thoracic aorta. Interestingly, the novel ERK‐selective inhibitor, FR18024, greatly attenuated the contractile effect of PE (*p *< 0.0001 for 0.1 μM and higher concentrations) in aortas from young mice (Figure 2A, left panel). FR18024 also reduced contractility of aged mouse aorta (*p* = 0.0047 for 0.5 μM; *p* = 0.0017 for 1 μM; *p *= 0.0005 for 5 µM; *p *= 0.0004 for 10µM), but the absolute decrease was much smaller due to the effect of ageing to diminish the control response to PE (Figure 2A, right panel). To further probe the effect of ageing on ERK activation, we conducted western blotting on protein samples from young and aged mouse aortas stimulated with PE (10 μM). Compared with young mice (2‐fold increase), PE‐induced ERK phosphorylation in aged mice (Figure 2B; 1.4‐fold increase) was decreased (*p *< 0.0001).

### 3.3 Increased miR‐34a and miR‐137 expression downregulates the expression of focal adhesion proteins in A7r5 cells and in aortic tissue, leading to defective ERK signalling

The question arises as to what causes the attenuation of ERK activation in the mouse aorta with increased age. We have previously demonstrated that miR‐34a and miR‐137 levels are increased with ageing and are predicted, by theoretical analyses, to target multiple genes that have been suggested to play a role in ERK signalling.^14^ Specifically, these miRs were predicted by computational methods (miRbase and TargetScan) to target the non‐receptor tyrosine kinase Src (miR‐34a and −137), the focal adhesion proteins Paxillin (miR‐34a and −137) and FAK (miR‐34a). To modify miR expression here, we transfected with miR mimics for −34a and −137 to enhance their activity in A7r5 smooth muscle cells. Smooth muscle cell lysates were harvested for measurement of gene expression (after 24 h) and protein (after 72 h) analysis by qPCR and western blotting, respectively.

**FIGURE 3:**
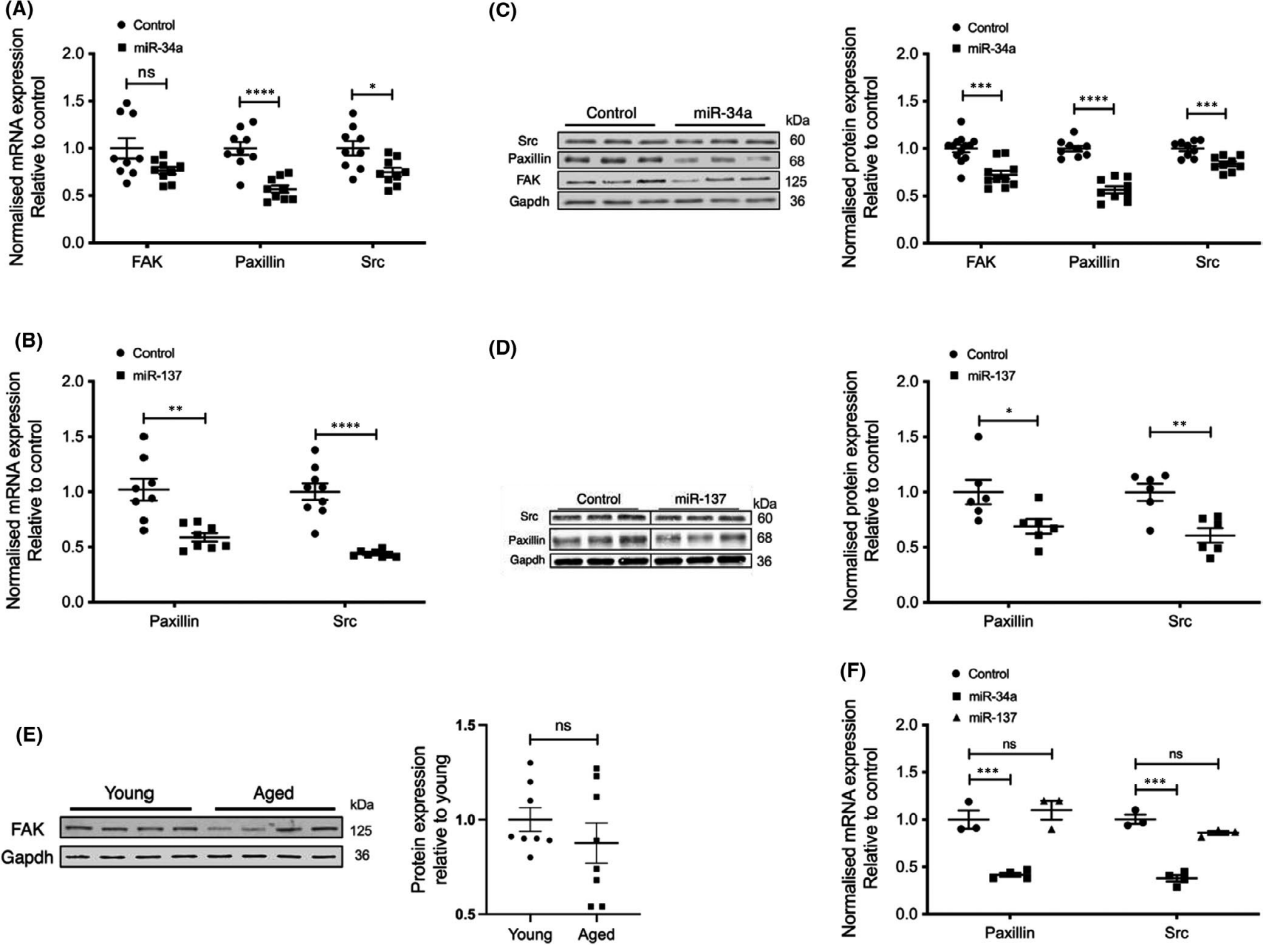
Overexpression of miR‐34a and miR‐137 downregulates focal adhesion proteins in A7r5 smooth muscle cells and in aortic tissue. Gene expression levels for the predicted targets of miR‐34a (A; *n* = 9 for each protein, control and miR‐34a) and miR‐137 (B; *n* = 8 for Pax, *n* = 9 for Src, control and miR‐137) were compared in A7r5 smooth muscle cells transfected with miR‐mimic or scrambled miR (control). Protein expression levels for the predicted targets of miR‐34a (C; *n* = 13 for FAK control, 10 for FAK plus miR‐34a; *n* = 9 for Pax control and Pax plus miR‐34a; *n* = 9 for Src control and Src plus miR‐34a) and miR‐137 (D; *n* = 6 for each protein, control and miR‐137) were compared in A7r5 cells transfected with miR‐mimic or scrambled miR (control). Black dividing lines indicate splicing of Western blot images for presentation. E, FAK protein levels were similar in aortas from young (*n* = 8) and aged (*n* = 8) mice. Representative Western blots are shown on the left and normalized data on the right. F, Gene expression levels for the predicted targets of miR‐34a (*n* = 4) and miR‐137 (*n* = 3) were compared in aortic smooth muscle tissues transfected with miR‐mimic or scramble miR (control). ****p < 0.0001, ***p < 0.001, **p < 0.01, *p < 0.05 (two‐tailed Student's *t* test)

The miR‐34a mimic induced decreases in Src (*p *= 0.0115) and paxillin (*p *< 0.0001) mRNA levels in the smooth muscle cells (Figure 3A). There was also a slight decrease in FAK mRNA expression (*p *= 0.0545), but this trend was not significant. Protein expression of Src (*p *= 0.0005), paxillin (*p *< 0.0001) and FAK (*p *= 0.0001) were also significantly downregulated 72 h after transfection with miR‐34a mimic (Figure 3C). Similarly, miR‐137 caused reduction of both mRNA and protein levels of Src (*p *< 0.0001 for mRNA decrease; *p *= 0.0034 for protein decrease) and paxillin (*p *= 0.0012 for mRNA decrease; *p *= 0.0364 for protein decrease) (Figure 3B,D respectively). These findings indicate that miR‐34a and −137 regulate the expression of important proteins previously associated with ERK signalling and also those associated with remodelling of focal adhesions in smooth muscle cells.

We have previously shown that the expression of the focal adhesion proteins Src and paxillin are downregulated by ageing in the mouse aorta.^14^ Here, we assessed the effect of ageing on FAK protein expression. As shown in Figure 3E, ageing did not significantly influence FAK expression.

To further determine whether similar results can be obtained in actual aortic tissue, we transfected miR‐34 and −137 mimic into freshly dissected aortic tissue rings. The aortic tissues were incubated for 48 h in optimem/Hanks and then used to extract RNA for gene analyses. We observed significant decreases in mRNA levels of Src (*p *= 0.0001) and paxillin (*p *= 0.0009) in aortic tissues transfected with miR‐34 mimic, while no change was seen (*p *= 0.0548 for Src and *p *= 0.5087 for paxillin) in miR‐137‐transfected tissues (Figure 3F). We also attempted to measure the expected corresponding changes in protein expression of Src and paxillin in transfected live aorta. However, the tissues did not live significantly longer than 72 h which was not long enough to detect protein changes. Thus, miR‐34a appears to be more important in the regulation of gene expression of Src and paxillin.

**FIGURE 4:**
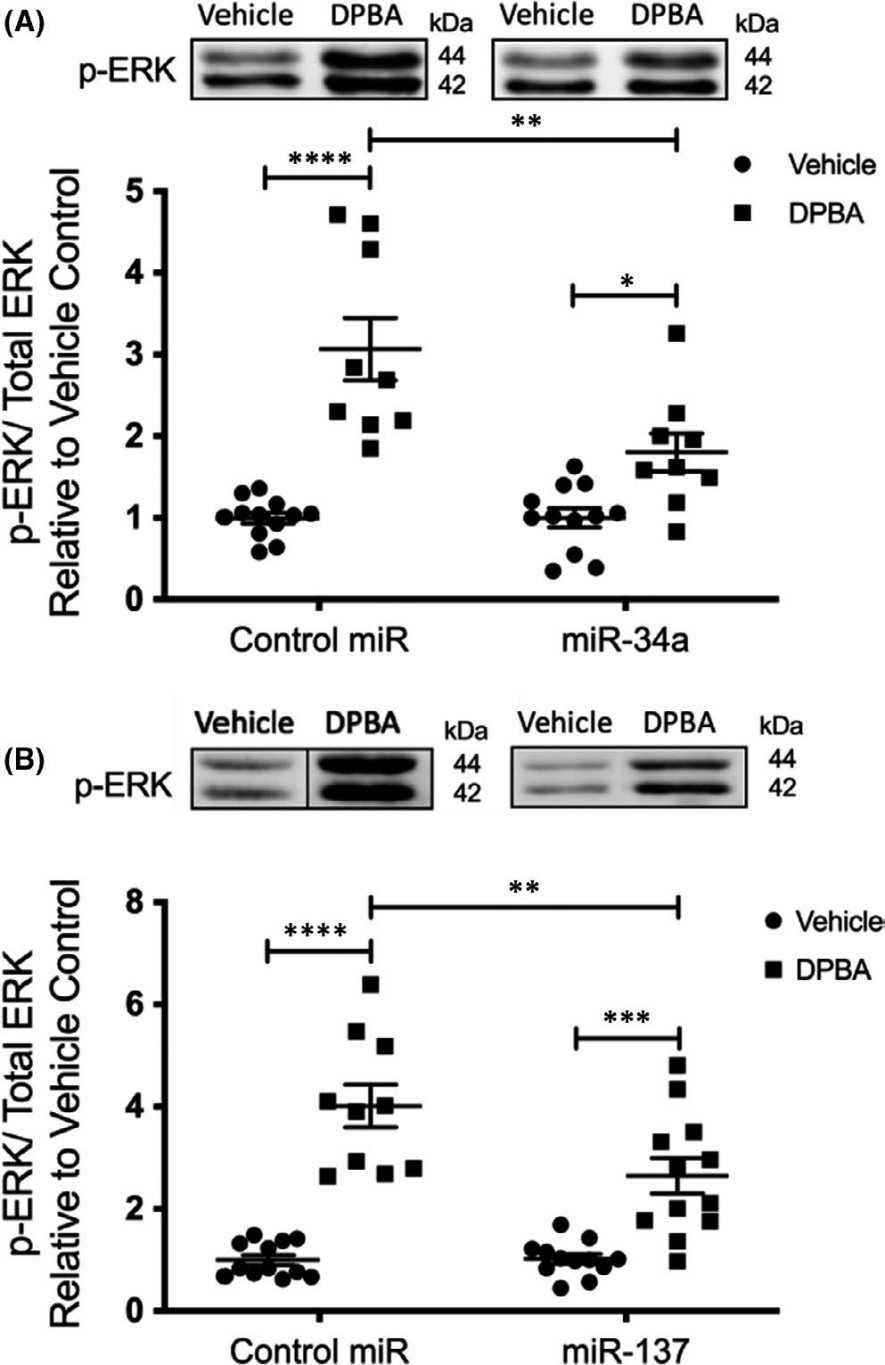
miR‐34a and miR‐137 overexpression attenuate agonist‐induced ERK activation in aortic smooth muscle cells. DPBA‐induced ERK phosphorylation in A7r5 smooth muscle cells transfected with mimics for miR‐34a (A; *n* = 12 both vehicle controls, *n* = 9 both DPBA‐treated) or miR‐137 (B; *n* = 12 both vehicle controls, *n* = 10 DPBA‐treated plus scrambled miR and *n* = 12 DPBA‐treated plus miR‐137) was reduced compared to control mimic‐treated cells. Black dividing lines indicate splicing of Western blot images for presentation. ****p < 0.0001, ***p < 0.001, **p < 0.01, *p < 0.05 (two‐way ANOVA)

To test the hypothesis that these two miRs can cause these age‐related defects in agonist‐induced ERK activation, we quantitated phorbol ester (DPBA, 12‐deoxyphorbol 13‐isobutyrate 20‐acetate)‐induced ERK 1/2 phosphorylation in miR mimic‐transfected A7r5 cells after 72‐h incubation. As shown in Figure 4, both miR‐34a (Figure 4A; *p *= 0.0014) and miR‐137 (Figure 4B; *p *= 0.0049) significantly decrease DPBA‐induced ERK activation in A7r5 smooth muscle cells.

### 3.4 Src and paxillin are required for agonist‐induced ERK activation

**FIGURE 5:**
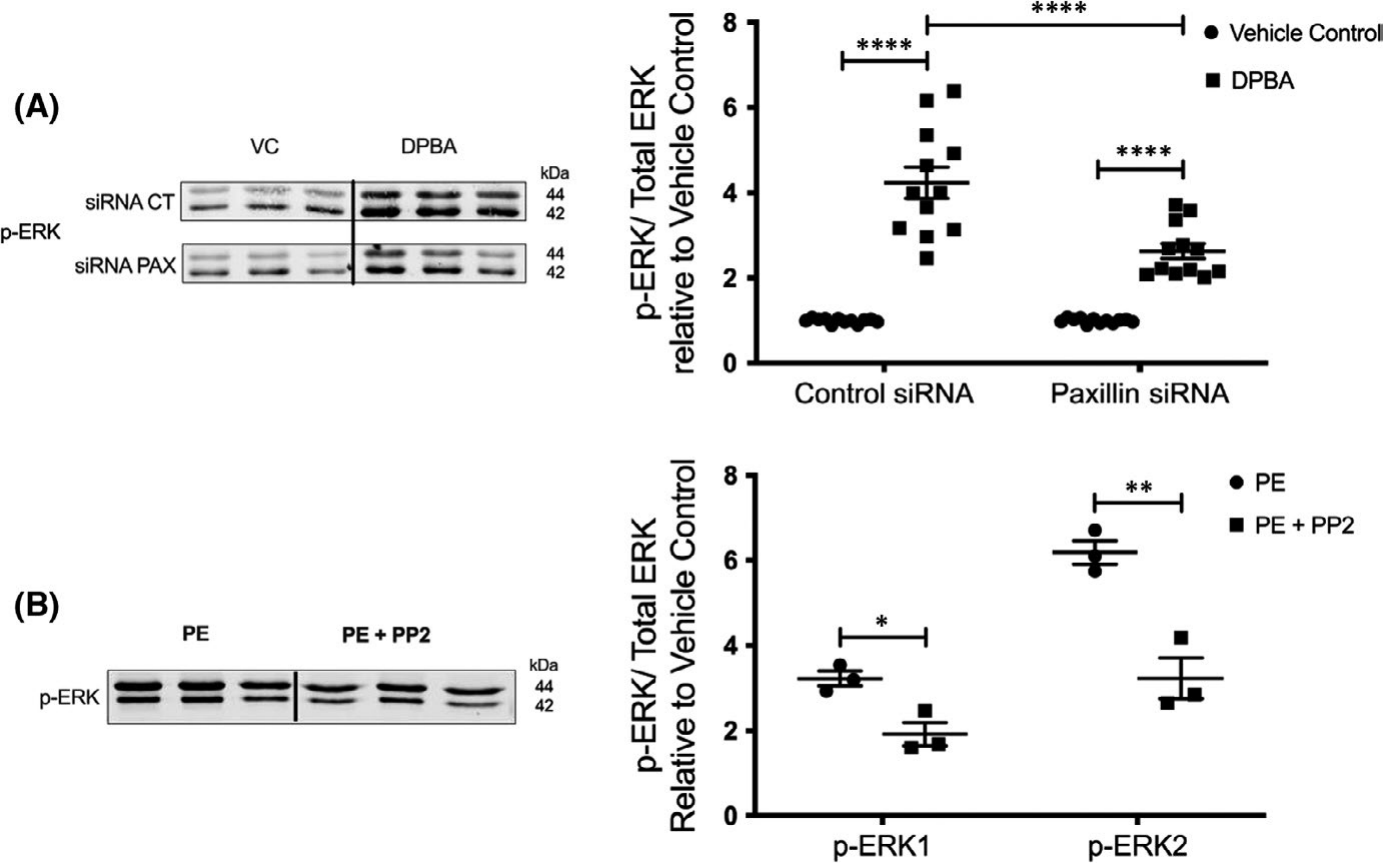
Src and paxillin play critical roles in agonist‐induced ERK activation in aortic smooth muscle cells. A, DPBA‐induced ERK phosphorylation in A7r5 cells transfected with Paxillin siRNA (*n* = 12) was blunted compared to CTRL siRNA‐treated cells (*n* = 12) (two‐way ANOVA). B, PE‐induced ERK phosphorylation in proximal aortic tissue strips pre‐treated with either PP2 (Src inhibitor, 10 µM; *n* = 3) or vehicle control (H_2_O; *n* = 3). PP2 pre‐treatment abrogated PE‐induced ERK phosphorylation (two‐tailed Student's *t* test). ****p < 0.0001, ***p < 0.001, **p < 0.01, *p < 0.05

Having determined that miR‐34a and miR‐137 downregulate Src and paxillin in A7r5 cells, we next sought to investigate whether Src and paxillin play roles in effecting the diminished agonist‐induced ERK signalling caused by these miRs. We therefore tested whether interfering with Src or paxillin influences agonist‐induced ERK activation. As shown in Figure 5A, knocking down paxillin in A7r5 cells using paxillin siRNA attenuated DPBA‐induced ERK activation (*p *< 0.0001). Similarly, the use of PP2, a Src inhibitor, significantly reduces PE‐induced ERK phosphorylation in aortic tissue (Figure 5B; *p *= 0.0158 for ERK1 and *p *= 0.0061 for ERK2), indicating that Src is required for ERK phosphorylation.

**FIGURE 6:**
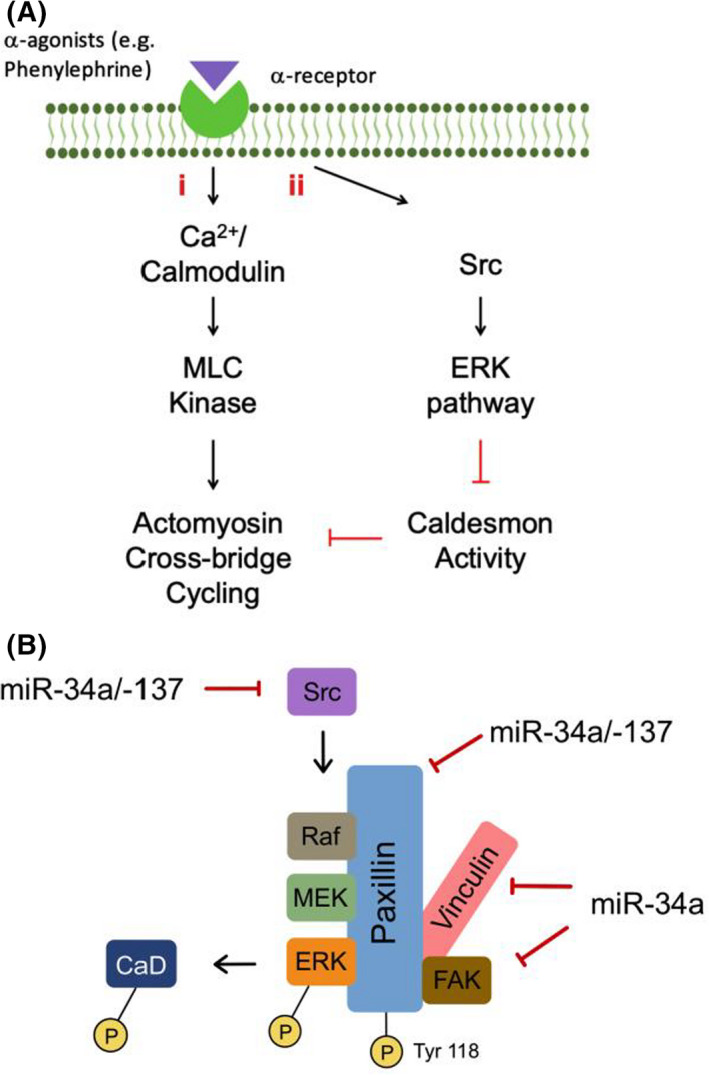
Summary diagram/Graphical Abstract. A, Mechanisms of ERK‐induced smooth muscle cell contraction with ageing. (i) MLC Kinase is a Ca^2+^/Calmodulin‐dependent kinase and can be activated by increases in cytoplasmic ionized calcium ([Ca^2+^i]) levels, induced by depolarization or G‐protein‐coupled receptor‐mediated agonists, such as alpha adrenergic (α) agonists. (ii) agonists have also been demonstrated to promote the PKC‐dependent activation of MEK that subsequently phosphorylates ERK, resulting in its activation. ERK activation induces phosphorylation of the actin‐binding protein caldesmon, which releases its inhibitory action on actin, resulting in enhanced actomyosin cross‐bridge cycling. B, Diagrammatic summary of the effects of miR‐34a and −137 on ERK signalling. These miRs reduce the expression of both activators of and scaffolding proteins for ERK signalling. Src activates ERK through the direct phosphorylation of Raf.^19^, ^20^ Paxillin functions as a scaffolding protein for the organization and activation of the ERK‐activating proteins FAK, Raf and MEK at focal adhesions^21^

These findings indicate that the age‐related increase in miR‐34a and −137 contribute to the downregulation of Src and paxillin protein levels, which promote aortic smooth muscle contractile dysfunction through a decrease in agonist‐induced ERK signalling (Summarized in Figure 6A,B).

## 4 DISCUSSION

In the current study, we discovered that ageing attenuates contractility of the mouse thoracic aorta and investigated the molecular mechanisms involved. We found that these mechanisms involve a microRNA‐induced downregulation of ERK pathway proteins. We provide evidence that agonist‐induced ERK signalling, which is decreased with ageing in the mouse aorta, is required for effective contractile function and a healthy aortic phenotype. Ageing increased the expression of miR‐34a and miR‐137, which led to the downregulation of their targets including paxillin and Src. We also demonstrate that overexpression of these miRs blunts agonist‐induced ERK activation. Finally, we show that both Src and paxillin are necessary for proper agonist‐induced ERK signalling in aortic smooth muscle cells. Importantly, the disruption of contractile signalling pathways with age may contribute to aortic pathophysiologies such as aneurysm formation and aortic dissection.

In the current study, we demonstrate, for the first time, that alpha agonist‐induced ERK activation is diminished with ageing. The impairment of ERK signalling is likely caused by the age‐related increase in microRNAs. We show here that miR‐34a and miR‐137 impair agonist‐induced ERK signalling through the targeted downregulation of Src and paxillin. Previous studies have suggested a role for these proteins in ERK activation in other non‐vascular cell types. For example, Src regulates ERK activation in neuronal cells via direct phosphorylation of Raf‐1 at its Tyr 340/341.^19^, ^20^ In addition, paxillin has been demonstrated to act as a scaffold for the organization and activation of the proteins Raf, MEK and ERK at focal adhesions,^21^ although the present study is the first study to do so in vascular smooth muscle cells. We also show here that PE‐induced ERK activation is Src dependent in vascular smooth muscle, supporting a previous study in the portal vein of the ferret.^22^ Additionally, we demonstrate, for the first time, that agonist‐induced ERK phosphorylation is diminished by paxillin knockdown in aortic smooth muscle cells.

The regulation of VSMC contraction has been shown to involve the activation of ERK, which phosphorylates and inactivates the actin‐binding protein caldesmon, alleviating caldesmon‐induced inhibition of myosin‐actin interactions, thus promoting VSMC contraction.^22^, ^23^, ^24^, ^25^, ^26^ Our data show that miR‐related degradation of Src and paxillin can impair the activity of ERK and thus promote a weakened contractile response to the agonist phenylephrine. Our finding that ageing promotes smooth muscle contractile dysfunction of the mouse aorta is in agreement with previously published data from other groups.^27^, ^28^ It has been reported that a compromised ability of vascular smooth muscle cells to contract underlies thoracic aortic aneurysm disease (TAAD).^27^, ^28^, ^29^, ^30^, ^31^, ^32^, ^33^, ^34^ We have directly shown that agonist‐induced ERK and focal adhesion remodelling pathways^35^ are impaired in the aged mouse aorta, which results in a reduced ability of the smooth muscle to contract. Since TAAD risk increases in the elderly,^36^ our data provide novel mechanistic insights into how the aged aorta may become susceptible to this pathology. It is to be noted that the sort of direct in vitro biomechanical, biochemical and molecular alterations observed here in the mouse aorta model have not yet been reported for comparable aged human aortas undoubtedly due to the lack of ready availability of such human tissues.

Interestingly, some previous studies have reported an increase in ERK phosphorylation in mouse models of TAAD.^37^, ^38^ It is possible that basal ERK phosphorylation is higher in aged mice, but that the downregulation of Src, paxillin and MEK reduces the ability of ERK to be activated under hemodynamic stresses. Alternatively, different pools of ERK could be involved. Importantly, the aforementioned studies suggested the use of pharmaceutical agents to blunt ERK activation in the treatment of TAAD.^22^, ^37^, ^38^ However, our results would argue against this approach. As we and others have shown ERK activation to be important for smooth muscle contraction^22^, ^24^, ^25^, ^39^ such agents will likely weaken aortic contractile function. Thus, since smooth muscle contractile dysfunction appears to be a key component of TAAD progression,^40^ our data suggest that pharmaceutical manipulation of ERK signalling should be approached with caution.

Previous studies have elucidated an important role for focal adhesion signalling in the development and regulation of vascular tone.^41^, ^42^ Focal adhesions (FAs) are dynamic structures that physically connect the actin cytoskeleton to the extracellular matrix (ECM) and have been proposed to play a role in aneurysm development.^40^ Focal adhesion signalling and remodelling is initiated by Src‐dependent tyrosine phosphorylation of the FA proteins paxillin, CAS and FAK.^43^, ^44^, ^45^, ^46^ We now show here that, along with miR‐203,^14^ miR‐34a and miR‐137 contribute to the age‐related decrease in Src and paxillin expression in A7r5 cells. We also, importantly, show that miR‐34a also has this effect on gene expression of Src and paxillin in actual contractile tissues. Despite a known role for FAK in ERK signalling,^47^ we demonstrate here that FAK protein levels are not significantly regulated by ageing, suggesting it is not responsible for this aged phenotype.

Thus, we propose that in the aged aorta, the mechanisms that lead to ERK activation are impaired, leading to a compromised contractile response of the aortas from aged male C57BL/6J mice. Furthermore, the ageing‐associated increased expression of miR‐34a and miR‐137, contributes to a decrease in Src and paxillin protein expression. We provide evidence that Src and paxillin are required for effective agonist‐induced ERK activation in contractile aortic smooth muscle cells and thus that increased miR‐34a and miR‐137 impairs ERK signalling (Figure 6A,B).

In conclusion, this study provides novel insights into the molecular mechanisms of age‐related impairment of VSMC signalling and contractile function. It is well known that aortic stiffness increases with age but contractility of the aged aorta has been less well studied. A compromised ability of vascular smooth muscle cells to contract is thought to underlie thoracic aortic aneurysm and aortic dissection. We report here that impaired contractility of the proximal aorta occurs with age and is agonist‐specific. Furthermore, we show that impaired aortic contractility is characteristic of a specific miR‐34a and miR‐137‐driven impairment of focal adhesion signalling and ERK activation. These molecular changes are potentially therapeutically addressable and hence of considerable importance. We suggest that future studies might utilize these findings to create novel miR‐targeting therapeutic strategies that could prevent aortic dysfunction in the elderly.

## CONFLICT OF INTEREST

None.

## AUTHOR CONTRIBUTION

**Christopher Nicholson:** Conceptualization (equal); Data curation (equal); Formal analysis (equal); Investigation (equal); Methodology (equal); Validation (equal); Writing – original draft (equal); Writing – review & editing (equal). **Yi Xing:** Conceptualization (equal); Data curation (equal); Formal analysis (equal); Investigation (equal); Methodology (equal); Validation (equal); Visualization (equal); Writing – review & editing (equal). **Sophie Lee:** Formal analysis (equal); Investigation (equal); Validation (equal). **Stephanie Liang:** Formal analysis (equal); Investigation (equal); Validation (equal). **Shivani Mohan:** Data curation (equal); Formal analysis (equal); Investigation (equal); Validation (equal). **Caitlin O'Rourke:** Formal analysis (equal); Investigation (equal); Validation (equal). **Joshua Kang:** Data curation (equal); Formal analysis (equal); Investigation (equal); Validation (equal). **Kathleen G. Morgan:** Conceptualization (equal); Funding acquisition (equal); Methodology (equal); Project administration (equal); Resources (equal); Supervision (equal); Writing – original draft (equal); Writing – review & editing (equal).

## Data Availability

The data that support the findings of this study are available from the corresponding author upon reasonable request.

## References

[1] Brozovich FV , Nicholson CJ , Degen CV , Gao YZ , Aggarwal M , Morgan KG . Mechanisms of vascular smooth muscle contraction and the basis for pharmacologic treatment of smooth muscle disorders. Pharmacol Rev. 2016;68:476‐532. 27037223 10.1124/pr.115.010652 PMC4819215

[2] Li Y , Je HD , Malek S , Morgan KG . ERK1/2‐mediated phosphorylation of myometrial caldesmon during pregnancy and labor. Am J Physiol Regul Integr Comp Physiol. 2003;284:R192‐199. 12388473 10.1152/ajpregu.00290.2002

[3] Li Y , Je HD , Malek S , Morgan KG . Role of ERK1/2 in uterine contractility and preterm labor in rats. Am J Physiol Regul Integr Comp Physiol. 2004;287:R328‐335. 15072963 10.1152/ajpregu.00042.2004

[4] Xiao D , Zhang L . ERK MAP kinases regulate smooth muscle contraction in ovine uterine artery: effect of pregnancy. Am J Physiol Heart Circ Physiol. 2002;282:H292‐300. 11748074 10.1152/ajpheart.2002.282.1.H292

[5] Anderson NG , Maller JL , Tonks NK , Sturgill TW . Requirement for integration of signals from two distinct phosphorylation pathways for activation of MAP kinase. Nature. 1990;343:651‐653. 2154696 10.1038/343651a0

[6] Bryan J . Caldesmon: fragments, sequence, and domain mapping. Ann N Y Acad Sci. 1990;599:100‐110. 2221667 10.1111/j.1749-6632.1990.tb42368.x

[7] Sobue K , Morimoto K , Kanda K , Maruyama K , Kakiuchi S . Reconstitution of Ca^2+^‐sensitive gelation of actin filaments with filamin, caldesmon and calmodulin. FEBS Lett. 1982;138:289‐292. 7067839 10.1016/0014-5793(82)80463-8

[8] Wang CL , Wang LW , Xu SA , Lu RC , Saavedra‐Alanis V , Bryan J . Localization of the calmodulin‐ and the actin‐binding sites of caldesmon. J Biol Chem. 1991;266:9166‐9172. 2026616

[9] Nemoto K , Vogt A , Oguri T , Lazo JS . Activation of the Raf‐1/MEK/Erk kinase pathway by a novel Cdc25 inhibitor in human prostate cancer cells. Prostate. 2004;58:95‐102. 14673957 10.1002/pros.10292

[10] Ok SH , Kwon SC , Yeol Han J , et al. Mepivacaine‐induced contraction involves increased calcium sensitization mediated via Rho kinase and protein kinase C in endothelium‐denuded rat aorta. Eur J Pharmacol. 2014;723:185‐193. 24333215 10.1016/j.ejphar.2013.11.040

[11] Streefkerk JO , Hoogaars WM , Christoffels VM , et al. Vasopressin‐induced vasoconstriction is dependent on MAPKerk1/2 phosphorylation. Fundam Clin Pharmacol. 2004;18:45‐50. 14748753 10.1046/j.1472-8206.2003.00221.x

[12] Zhao Z , Wang J , Huo Z , Wang Z , Mei Q . FTY720 elevates smooth muscle contraction of aorta and blood pressure in rats via ERK activation. Pharmacol Res Perspect. 2017;5:e00308. 28480040 10.1002/prp2.308 PMC5415948

[13] Zhou J , Li C , Gu G , Wang Q , Guo M . Selenoprotein N was required for the regulation of selenium on the uterine smooth muscle contraction in mice. Biol Trace Elem Res. 2018;183:138‐146. 28836095 10.1007/s12011-017-1130-z

[14] Nicholson CJ , Seta F , Lee S , Morgan KG . MicroRNA‐203 mimics age‐related aortic smooth muscle dysfunction of cytoskeletal pathways. J Cell Mol Med. 2017;21:81‐95. 27502584 10.1111/jcmm.12940 PMC5192880

[15] Marganski WA , Gangopadhyay SS , Je HD , Gallant C , Morgan KG . Targeting of a novel Ca^2+^/calmodulin‐dependent protein kinase II is essential for extracellular signal‐regulated kinase‐mediated signaling in differentiated smooth muscle cells. Circ Res. 2005;97:541‐549. 16109919 10.1161/01.RES.0000182630.29093.0d

[16] Kimes BW , Brandt BL . Characterization of two putative smooth muscle cell lines from rat thoracic aorta. Exp Cell Res. 1976;98:349‐366. 943301 10.1016/0014-4827(76)90446-8

[17] Livak KJ , Schmittgen TD . Analysis of relative gene expression data using real‐time quantitative PCR and the 2(‐Delta Delta C(T)) Method. Methods. 2001;25:402‐408. 11846609 10.1006/meth.2001.1262

[18] Dessy C , Kim I , Sougnez CL , Laporte R , Morgan KG . A role for MAP kinase in differentiated smooth muscle contraction evoked by alpha‐adrenoceptor stimulation. Am J Physiol. 1998;275:C1081‐1086. 9755061 10.1152/ajpcell.1998.275.4.C1081

[19] Dasgupta P , Rastogi S , Pillai S , et al. Nicotine induces cell proliferation by beta‐arrestin‐mediated activation of Src and Rb‐Raf‐1 pathways. J Clin Invest. 2006;116:2208‐2217. 16862215 10.1172/JCI28164 PMC1513051

[20] Tian HP , Huang BS , Zhao J , Hu XH , Guo J , Li LX . Non‐receptor tyrosine kinase Src is required for ischemia‐stimulated neuronal cell proliferation via Raf/ERK/CREB activation in the dentate gyrus. BMC Neurosci. 2009;10:139. 19943942 10.1186/1471-2202-10-139 PMC2794287

[21] Ishibe S , Joly D , Liu ZX , Cantley LG . Paxillin serves as an ERK‐regulated scaffold for coordinating FAK and Rac activation in epithelial morphogenesis. Mol Cell. 2004;16:257‐267. 15494312 10.1016/j.molcel.2004.10.006

[22] Saphirstein RJ , Gao YZ , Lin QQ , Morgan KG . Cortical actin regulation modulates vascular contractility and compliance in veins. J Physiol. 2015;593:3929‐3941. 26096914 10.1113/JP270845 PMC4575578

[23] Somlyo AP , Somlyo AV . Ca2+ sensitivity of smooth muscle and nonmuscle myosin II: modulated by G proteins, kinases, and myosin phosphatase. Physiol Rev. 2003;83:1325‐1358. 14506307 10.1152/physrev.00023.2003

[24] Li Y , Gallant C , Malek S , Morgan KG . Focal adhesion signaling is required for myometrial ERK activation and contractile phenotype switch before labor. J Cell Biochem. 2007;100:129‐140. 16888778 10.1002/jcb.21033

[25] Li Y , Reznichenko M , Tribe RM , et al. Stretch activates human myometrium via ERK, caldesmon and focal adhesion signaling. PLoS One. 2009;4:e7489. 19834610 10.1371/journal.pone.0007489 PMC2759504

[26] Gerthoffer WT . Signal‐transduction pathways that regulate visceral smooth muscle function. III. Coupling of muscarinic receptors to signaling kinases and effector proteins in gastrointestinal smooth muscles. Am J Physiol Gastrointest Liver Physiol. 2005;288:G849‐853. 15826932 10.1152/ajpgi.00530.2004

[27] Wheeler JB , Mukherjee R , Stroud RE , Jones JA , Ikonomidis JS . Relation of murine thoracic aortic structural and cellular changes with aging to passive and active mechanical properties. J Am Heart Assoc. 2015;4:e001744. 25716945 10.1161/JAHA.114.001744 PMC4392448

[28] Wang M , Fu Y , Gao C , et al. Cartilage oligomeric matrix protein prevents vascular aging and vascular smooth muscle cells senescence. Biochem Biophys Res Commun. 2016;478:1006‐1013. 27498005 10.1016/j.bbrc.2016.08.004

[29] Murata T , Lin MI , Huang Y , et al. Reexpression of caveolin‐1 in endothelium rescues the vascular, cardiac, and pulmonary defects in global caveolin‐1 knockout mice. J Exp Med. 2007;204:2373‐2382. 17893196 10.1084/jem.20062340 PMC2118452

[30] Milewicz DM , Trybus KM , Guo DC , et al. Altered smooth muscle cell force generation as a driver of thoracic aortic aneurysms and dissections. Arterioscler Thromb Vasc Biol. 2017;37:26‐34. 27879251 10.1161/ATVBAHA.116.303229 PMC5222685

[31] Milewicz DM , Guo DC , Tran‐Fadulu V , et al. Genetic basis of thoracic aortic aneurysms and dissections: focus on smooth muscle cell contractile dysfunction. Annu Rev Genomics Hum Genet. 2008;9:283‐302. 18544034 10.1146/annurev.genom.8.080706.092303

[32] Guo F , Li Z , Song L , et al. Increased apoptosis and cysteinyl aspartate specific protease‐3 gene expression in human intracranial aneurysm. J Clin Neurosci. 2007;14:550‐555. 17430778 10.1016/j.jocn.2005.11.018

[33] de Carcer G , Wachowicz P , Martinez‐Martinez S , et al. Plk1 regulates contraction of postmitotic smooth muscle cells and is required for vascular homeostasis. Nat Med. 2017;23:964‐974. 28692064 10.1038/nm.4364

[34] Chung AW , Au Yeung K , Sandor GG , Judge DP , Dietz HC , van Breemen C . Loss of elastic fiber integrity and reduction of vascular smooth muscle contraction resulting from the upregulated activities of matrix metalloproteinase‐2 and ‐9 in the thoracic aortic aneurysm in Marfan syndrome. Circ Res. 2007;101:512‐522. 17641224 10.1161/CIRCRESAHA.107.157776

[35] Gao YZ , Saphirstein RJ , Yamin R , Suki B , Morgan KG . Aging impairs smooth muscle‐mediated regulation of aortic stiffness: a defect in shock absorption function? Am J Physiol Heart Circ Physiol. 2014;307:H1252‐1261. 25128168 10.1152/ajpheart.00392.2014 PMC4200340

[36] Pisano C , Balistreri CR , Ricasoli A , Ruvolo G . Cardiovascular disease in ageing: an overview on thoracic aortic aneurysm as an emerging inflammatory disease. Mediators Inflamm. 2017;2017:1274034. 29203969 10.1155/2017/1274034 PMC5674506

[37] Yang P , Schmit BM , Fu C , et al. Smooth muscle cell‐specific Tgfbr1 deficiency promotes aortic aneurysm formation by stimulating multiple signaling events. Sci Rep. 2016;6:35444. 27739498 10.1038/srep35444 PMC5064316

[38] Doyle JJ , Doyle AJ , Wilson NK , et al. A deleterious gene‐by‐environment interaction imposed by calcium channel blockers in Marfan syndrome. Elife. 2015;4. e08648. 26506064 10.7554/eLife.08648 PMC4621743

[39] Langlois B , Belozertseva E , Parlakian A , et al. Vimentin knockout results in increased expression of sub‐endothelial basement membrane components and carotid stiffness in mice. Sci Rep. 2017;7:11628. 28912461 10.1038/s41598-017-12024-z PMC5599644

[40] Humphrey JD , Schwartz MA , Tellides G , Milewicz DM . Role of mechanotransduction in vascular biology: focus on thoracic aortic aneurysms and dissections. Circ Res. 2015;116:1448‐1461. 25858068 10.1161/CIRCRESAHA.114.304936 PMC4420625

[41] Srinivasan R , Forman S , Quinlan RA , Ohanian J , Ohanian V . Regulation of contractility by Hsp27 and Hic‐5 in rat mesenteric small arteries. Am J Physiol Heart Circ Physiol. 2008;294:H961‐969. 18083901 10.1152/ajpheart.00939.2007

[42] Mills RD , Mita M , Walsh MP . A role for the Ca‐dependent tyrosine kinase Pyk2 in tonic depolarization‐induced vascular smooth muscle contraction. J Muscle Res Cell Motil. 2015;36:479–489. 26150074 10.1007/s10974-015-9416-2

[43] Zhang W , Huang Y , Wu Y , Gunst SJ . A novel role for RhoA GTPase in the regulation of airway smooth muscle contraction. Can J Physiol Pharmacol. 2015;93:129‐136. 25531582 10.1139/cjpp-2014-0388 PMC4399233

[44] Min J , Reznichenko M , Poythress RH , et al. Src modulates contractile vascular smooth muscle function via regulation of focal adhesions. J Cell Physiol. 2012;227:3585‐3592. 22287273 10.1002/jcp.24062 PMC3348426

[45] Chen S , Wang R , Li QF , Tang DD . Abl knockout differentially affects p130 Crk‐associated substrate, vinculin, and paxillin in blood vessels of mice. Am J Physiol Heart Circ Physiol. 2009;297:H533‐539. 19542491 10.1152/ajpheart.00237.2009 PMC2724200

[46] Anfinogenova Y , Wang R , Li QF , Spinelli AM , Tang DD . Abl silencing inhibits CAS‐mediated process and constriction in resistance arteries. Circ Res. 2007;101:420‐428. 17615370 10.1161/CIRCRESAHA.107.156463 PMC2084484

[47] Sawai H , Okada Y , Funahashi H , et al. Activation of focal adhesion kinase enhances the adhesion and invasion of pancreatic cancer cells via extracellular signal‐regulated kinase‐1/2 signaling pathway activation. Mol Cancer. 2005;4:37. 16209712 10.1186/1476-4598-4-37 PMC1266395

